# Visible-light driven degradation of tetracycline and ciprofloxacin using a Z-scheme FeS_2_@polythiophene heterostructure photocatalyst

**DOI:** 10.1039/d6na00587j

**Published:** 2026-09-14

**Authors:** Maruthasalam Pannerselvam, Vadivel Siva, Abdul Samad Shameem, Anbazhagan Murugan, Krishnasamy Padmavathi, Murugan Dineshkumar, Shaik Gouse Peera

**Affiliations:** a Department of Physics, Karpagam Academy of Higher Education Coimbatore 641 021 Tamil Nadu India siva33phy@gmail.com siva.vadivel@kahedu.edu.in; b Centre for Energy and Environment, Karpagam Academy of Higher Education Coimbatore 641 021 Tamil Nadu India; c Department of Science and Humanities, Karpagam Academy of Higher Education Coimbatore 641 021 Tamil Nadu India; d Department of Science and Humanities, Karpagam College of Engineering Coimbatore 641 032 Tamil Nadu India; e Department of Advanced Materials Engineering, College of Engineering, Keimyung University 1095 Dalgubeol-daero Daegu 42601 Republic of Korea gouse@kmu.ac.kr

## Abstract

The development of efficient and reusable visible-light-driven photocatalysts is essential for the sustainable removal of emerging pharmaceutical contaminants from wastewater. In this study, a FeS_2_@polythiophene (FeS_2_@PTh) heterostructure has been synthesized using a simple *in situ* chemical oxidative polymerization method. The synthesized FeS_2_@PTh was used as a visible-responsive photocatalyst for the degradation of tetracycline (TC) and ciprofloxacin (CIP) from an aqueous medium. XPS analysis confirmed the existence of Fe^2+^, Fe^3+^, and S 2p species, with a significant interface between the FeS_2_ and PTh systems. FeS_2_@PTh exhibited higher degradation performance, removing 83% of TC (*k* = 1.422 × 10^−2^ min^−1^) and 85% of CIP (*k* = 1.687 × 10^−2^ min^−1^) under visible-light irradiation. Moreover, this study highlighted the excellent photostability and reproducibility of the photocatalysts, contributing to the development of sustainable photocatalytic materials for efficient water treatment.

## Abstract 2

The FeS_2_@PTh nanohybrid exhibited high degradation of 83% of TC (*k* = 1.422 × 10^−2^ min^−1^) and 85% of CIP (*k* = 1.687 × 10^−2^ min^−1^) under visible-light irradiation.

## Graphical abstract

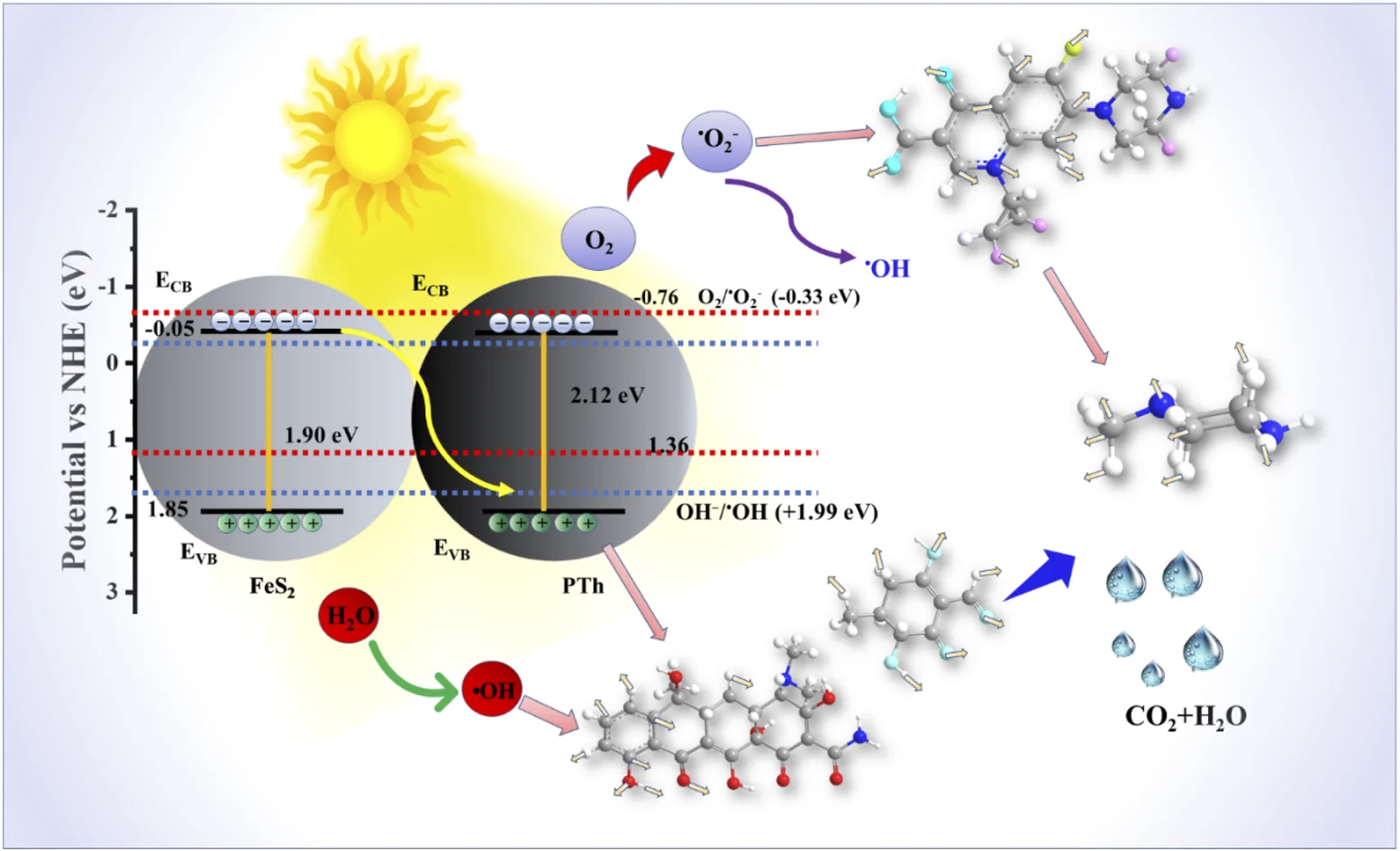

## 1. Introduction

Energy crises and environmental pollution remain key issues despite the rapid development in building a sustainable society.^1,2^ The aquatic ecosystem is severely contaminated by everyday effluents from industrial wastes, and agricultural wastes that are dumped into natural water bodies.^3,4^ In particular, pharmaceutical residues are new contaminants that pose a major environmental issue due to their toxicity and capacity to promote bacterial resistance.^5^ Many antibiotic medications, including ciprofloxacin and tetracycline, have been overused recently to treat fever, and bacterial infections.^6^ The occurrence of these antibiotics in the environment is primarily attributed to their discharge from pharmaceutical manufacturing industries, hospital wastes, and various industrial wastes, which enter wastewater streams and are frequently transferred into freshwater systems, such as lakes and rivers, where they accumulate in soil.^7^ Through these pathways, TC and CIP pollutants may re-enter the food chain, affecting human and animal health.^7–9^ The persistence of TC and CIP in aquatic environments poses a series of ecological and public health challenges. Owing to their stable chemical structures, these compounds resist natural biodegradation, leading to their accumulation in water bodies. Therefore, the removal of these pollutants from wastewater using conventional treatment methods often proves ineffective, highlighting the urgent need for advanced and efficient remediation strategies.^10–13^

Advanced oxidation processes (AOPs) have emerged as highly effective strategies for environmental remediation due to their ability to generate reactive species that can degrade persistent pollutants.^14^ Among these, heterogeneous photocatalysts have attracted considerable research interest owing to their sustainable development and efficiency in eliminating recalcitrant contaminants, ultimately breaking down and converting the pollutants into harmless and mineralised species using AOPs. Upon irradiation with light of sufficient energy, the electrons in the semiconductor photocatalyst are promoted from the valence band (VB) to the conduction band (CB), leaving behind positively charged holes (h^+^) in the VB. Photogenerated electron–hole pairs serve as a driving force that subsequently creates a redox mechanism on the catalyst surface.^15^ These photogenerated holes possess strong oxidative potential, reacting with adsorbed water molecules and hydroxyl ions (OH^−^) to produce highly reactive hydroxyl radicals (˙OH), simultaneously exciting electrons from the CB and reducing dissolved oxygen molecules to form superoxide radicals (˙O_2_^−^). These reactive species (ROS), along with (e^−^/h^+^) carriers, actively interact with pollutant molecules adsorbed on the photocatalyst surface. Through a sequence of oxidation and reduction reactions, complex organic contaminants are effectively broken down into simpler, non-toxic products, thereby completing the photocatalytic degradation process.^15–17^

To date, a wide range of visible-light responsive semiconductors have been extensively explored for energy conversion and environmental remediation strategies. Among them, metal chalcogenides, such as MoS_2_, WS_2_, FeS_2_ and ZnS, have gained significant attention due to their suitable bandgap energies, strong absorption capacity over the visible region, and tuneable electronic properties, making them promising candidates for applications in pollutant degradation, hydrogen production and wastewater treatment.^18^ Recent studies have demonstrated that FeS_2_ based photocatalysts exhibit remarkable efficiency in the degradation of TC and CIP pollutants. For instance, FeS_2_@Co_3_O_4_–C composites have achieved 97.2% removal of TC at 20 min under visible light, as reported by Wang *et al.*^19^ In a related study, Liu *et al.*^20^ demonstrated persulfate-activated FeS_2_ CIP removal, with the synergistic sulphur species facilitating regeneration of Fe^2+^ from Fe^3+^ to achieve a CIP removal efficiency of 94.4% within 60 min. FeS_2_/SiO_2_ microspheres activated *via* the hydrogen peroxide (H_2_O_2_) system show that removal of CIP could be achieved efficiently at 100% within 60 min, as reported by Diao *et al.*^21^ Mohanty *et al.* reported a poly-*m*-aminophenol/CoFe_2_O_4_ heterojunction that achieved nearly 100% mineralization of BPA and ciprofloxacin within 60 min under ambient conditions.^22^ A visible-light driven Ag_3_PO_4_–WO_3_/coffee bean biochar photocatalyst achieved nearly 100% mineralisation of CIP within 60 min.^23^ Mishra *et al.* developed a Z-scheme g-C_3_N_4_/Gd_2_O_3_ composite that achieved 97% degradation of CIP and 96.5% degradation of TC under visible light irradiation within 120 min. This catalyst demonstrates good recyclability over five consecutive cycles.^24^ Mishra *et al.*^25^ developed a g-C_3_N_4_/Sm_2_O_3_ photocatalyst that demonstrated 90.6% CIP within 120 min under visible light. This enhanced activity was attributed to synergistic factors that influence visible-light absorption.

Conductive polymers, such as polyaniline (PANI), polypyrrole (PPy), and polythiophene (PTh), possess π-conjugated structures that enable visible-light absorption and charge transport. However, they often suffer from rapid electron–hole recombination. Ahmad *et al.* reported ciprofloxacin removal efficiencies of 82% (PANI), 75% (PPy), and 70% (PTh) after 660 min of sunlight irradiation.^26^ In our previous study, a MoS_2_@PTh heterojunction achieved 90% TC and 75% CIP degradation, attributed to improved charge separation and enhanced reactive oxygen species generation.^27^ In this current study, an efficient nanoscale FeS_2_@PTh composite was prepared using a simple synthesis route. A comprehensive investigation was carried out to explore the morphological, structural and physico-chemical properties. The FeS_2_@PTh composite achieved efficient removal of TC (83%) and CIP (85%) within 120 min. The enhanced photocatalytic activity of the FeS_2_@PTh heterostructure is attributed to its efficient charge separation capability, facilitated by the formation of a Z-scheme system. This composite also exhibits excellent recyclability, making it a promising nanoscale photocatalyst. In this context, the Z-scheme charge-transfer pathway significantly improves the degradation of TC and CIP, where highly reactive hydroxyl radicals (˙OH) play a dominant role by attacking and decomposing the pollutant molecules.

## 2. Experimental section

### 2.1 Materials

Potassium persulphate (K_2_S_2_O_8_, 99.99%), potassium iodide (KI, 99.0%) and iron disulfide (FeS_2_, 99.8%) were purchased from Sigma-Aldrich. Thiophene (98%), isopropyl alcohol (C_3_H_8_O, 99%), and anhydrous ferric chloride (FeCl_3_, 98%) were obtained from Loba Chemie Pvt Ltd. Tetracycline hydrochloride and ciprofloxacin hydrochloride were purchased from HiMedia.

### 2.2 Synthesis of polythiophene and FeS_2_@PTh

Polythiophene was synthesised by a simple chemical oxidative polymerisation method. FeCl_3_ (4.866 g) was dissolved in 50 mL of chloroform and magnetically stirred for 20 min. Then, the monomer form of thiophene (0.793 mL) was added dropwise to the solution until a homogeneous mixture was obtained. The formation of a black precipitate was allowed to proceed for 10 h with constant stirring. After the complete process, the resultant PTh was washed with a 1 mol L^−1^ HCl solution to remove residual FeCl_3_ until a colourless product was obtained. Finally, the product was dried under vacuum at 60 °C overnight to obtain polythiophene. To synthesise FeS_2_@PTh nanocomposite an *in situ* chemical oxidative polymerisation was performed by adding 0.3 g of FeS_2_ NPs directly to the FeCl_3_ (4.866 g) solution. Afterwards, the thiophene monomer (0.793 mL) was added dropwise to the solution. The reaction was carried out for 6 h with constant stirring. After the complete process, the product was washed several times with 1 mol L^−1^ HCl solution to remove residual FeCl_3_ until a colourless product was obtained. The product was dried at 60 °C for 24 h to obtain FeS_2_@PTh. The preparation process of FeS_2_@PTh is shown in Fig. 1.

**Fig. 1:**
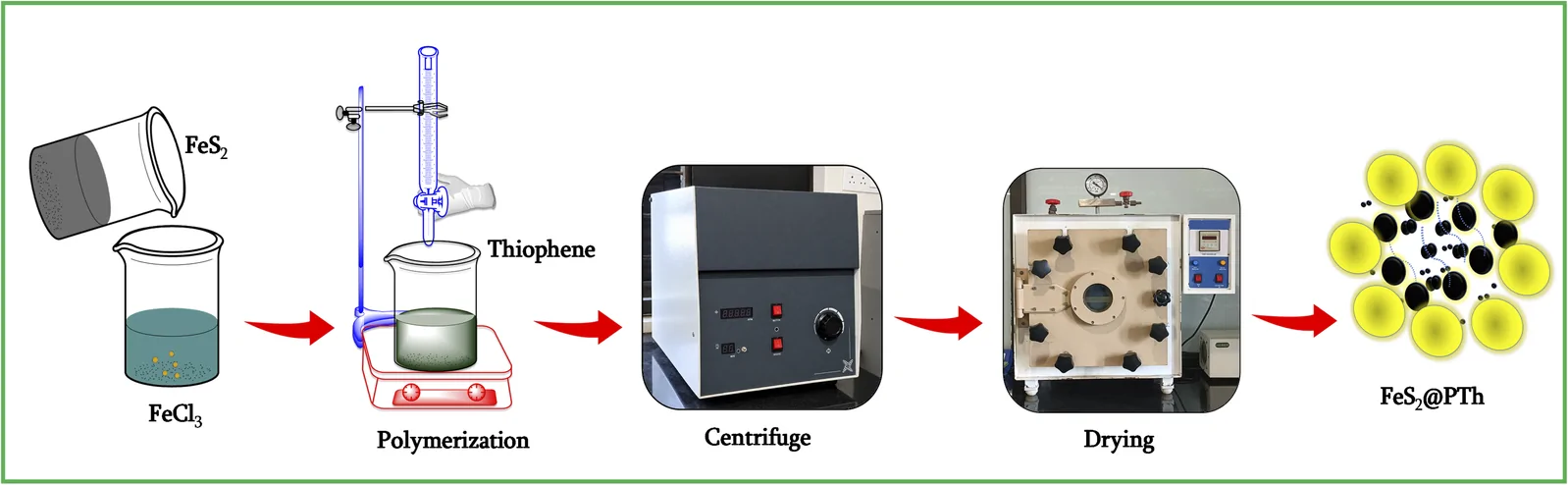
Schematic of the synthesis of the FeS_2_@PTh composite.

### 2.3 Photocatalytic degradation experiments

Photocatalytic degradation experiments were carried out under visible-light source. Stock solutions (1 × 10^−3^ M) of TC and CIP were prepared using distilled water. Further solutions were prepared by diluting 6 mL of TC and 3 mL of CIP stock solutions to a final volume of 100 mL, yielding concentrations of 6 × 10^−5^ M (∼26.7 mg L^−1^) and 3 × 10^−5^ M (∼10 mg L^−1^), respectively. The optimised standard stock result was verified using a UV-visible spectrophotometer. In a typical experiment, 30 mg of catalyst was dispersed in 100 mL of solution. Before light irradiation, the suspension was magnetically stirred in the dark condition for 30 min to establish adsorption/desorption equilibrium. Subsequently, the solution was exposed to 160 W illumination. At regular time intervals, the settled particle were collected, centrifuged to remove catalyst particles, and analysed.

### 2.4 Materials characterisation

X-ray diffraction patterns were recorded using a Bruker D8 ECO Advance with Cu K_α_ radiation (*λ* = 1.54 Å). The optical properties were recorded using a JASCO V-750 spectroscopy in the range of 200–600 nm. The surface morphology characteristics were analysed using a FESEM-Sigma HV (Carl Zeiss) equipped with a Bruker Quantax 200-Z10. HR-TEM was performed with a Philips-TEM CM20. Dynamic light scattering (Malvern Analytical Limited) was used for particle size analysis. X-ray photoelectron spectroscopy (XPS; Thermo Fisher Nexsa) was performed to evaluate the oxidation state and chemical composition. Photocatalytic activity was tested by HEBER Hml-Hvar-Compact-Lp-Mp46400 reactor.

## 3. Results and discussion

### 3.1 XRD analysis

The structural information of the synthesised FeS_2_@PTh was studied *via* X-ray diffraction (XRD). The XRD patterns are shown in Fig. 2 and display well-defined and sharp peaks for pristine FeS_2_ at 28.4°, 33.0°, 37.0°, 40.7°, 47.3°, 56.2°, 59.0°, 61.6°, 64.24°, 76.5° and 78.9° corresponding to the *hkl* planes (111), (200), (210), (211), (220), (311), (222), (230), (321), (331) and (420), respectively, which are notably consistent with the standard JCPDS card no.: 00-042-1340. These observations are consistent with FeS_2_. No traces of other phases are present, indicating the purity of the material.^28–30^ The diffraction patterns in Fig. 2 confirm the presence of FeS_2_ in the composite, while PTh shows amorphous phases within the 2*θ* range of 20°–30°, due to the disordered arrangement of polymer chains. Notably, the peaks for FeS_2_ in the composite clearly confirm the formation of FeS_2_@PTh, as PTh peaks are also obtained. Furthermore, the decrease in peak intensity of FeS_2_@PTh compared to FeS_2_ indicates that both PTh and FeS_2_ inhibit the growth of individual counterparts.

**Fig. 2:**
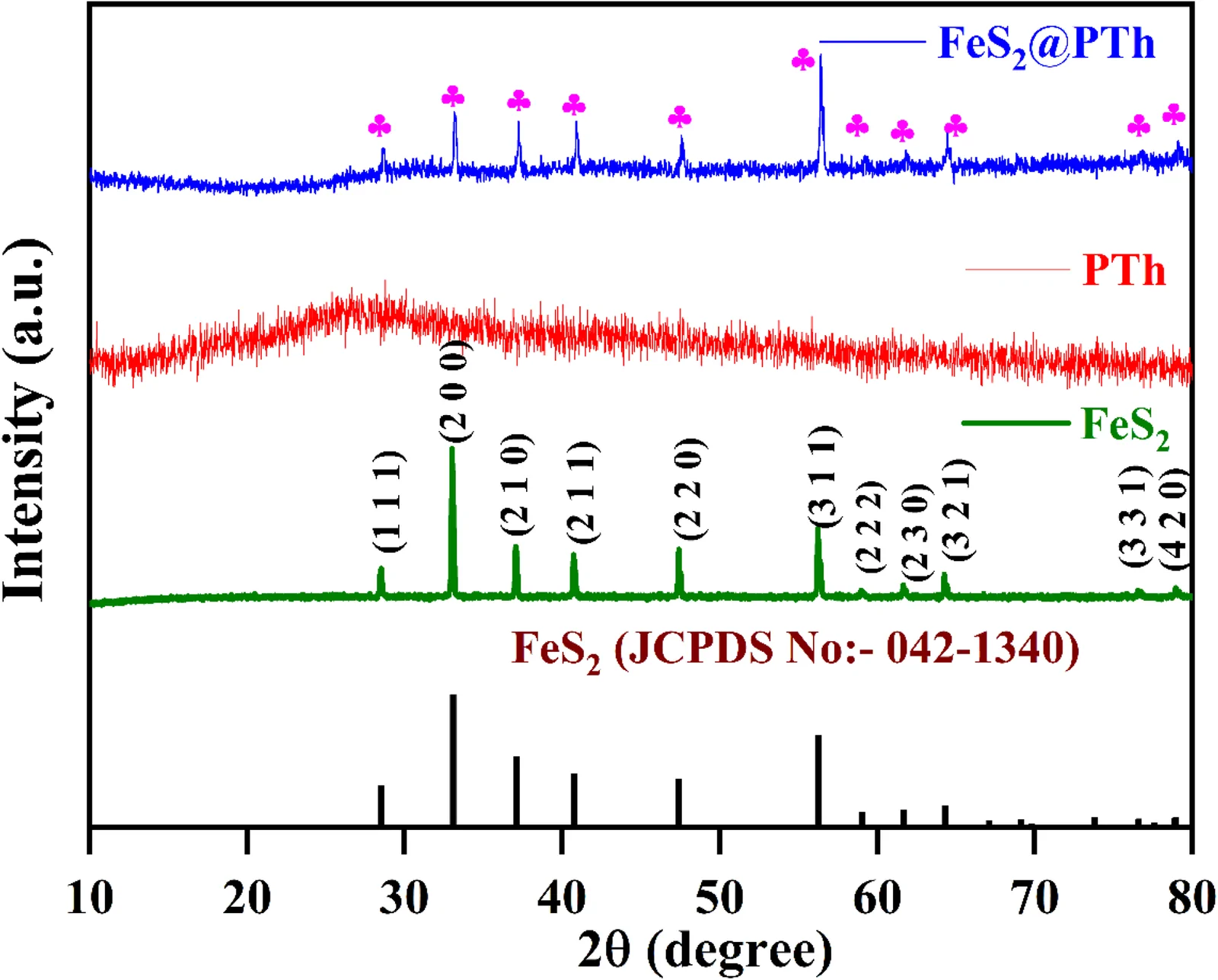
XRD patterns of PTh, FeS_2_ and FeS_2_@PTh.

### 3.2 XPS analysis

XPS analysis was conducted to investigate the chemical states of the composites and illustrated in Fig. 3. The XPS survey spectrum, shown in Fig. 3(a), confirms the presence of Fe, C, and S in the composite. The C 1s XPS spectrum in Fig. 3(b), calibrated using standardised carbon C 1s at 284.8 eV, was deconvoluted into three peaks at 284.8, 285.12, and 287.18 eV, corresponding to C–C/C=C, C–S, and C–O bonds originating from thiophene species.^27,31–33^ The S 2p XPS spectrum (Fig. 3(c)) displays binding energies at 162.56, 163.81, 165.31, 168.7, and 170.53 eV. The S 2p_3/2_ and S 2p_1/2_ signals at 162.56 and 163.81 eV are attributed to disulfide (S_2_^2−^) species in pyrite.^27,28,30,34^ The peak at 165.31 eV is assigned to C–S–C bonded polythiophene, indicating strong interaction between pyrite and the polymer matrix. Peaks at 168.7 and 170.52 eV are assigned to partially oxidised sulphur species, which are fitted with S 2p_3/2_ and S 2p_1/2_. The Fe 2p XPS spectrum (Fig. 3(d)) shows peaks at 710.4 and 723 eV, corresponding to Fe^2+^, confirming the presence of the FeS_2_ phase. Additional peaks at 711.4 and 724.5 eV are attributed to Fe^3+^, likely resulting from surface oxidation and interaction with the polymer.^35,36^ The prominent satellite peak at 719.1 eV further supports the existence of Fe^3+^ species.^37,38^ These results indicate that FeS_2_ in the FeS_2_@PTh composite is coordinated to C and S in the polymer matrix, demonstrating successful incorporation into the composite.

**Fig. 3:**
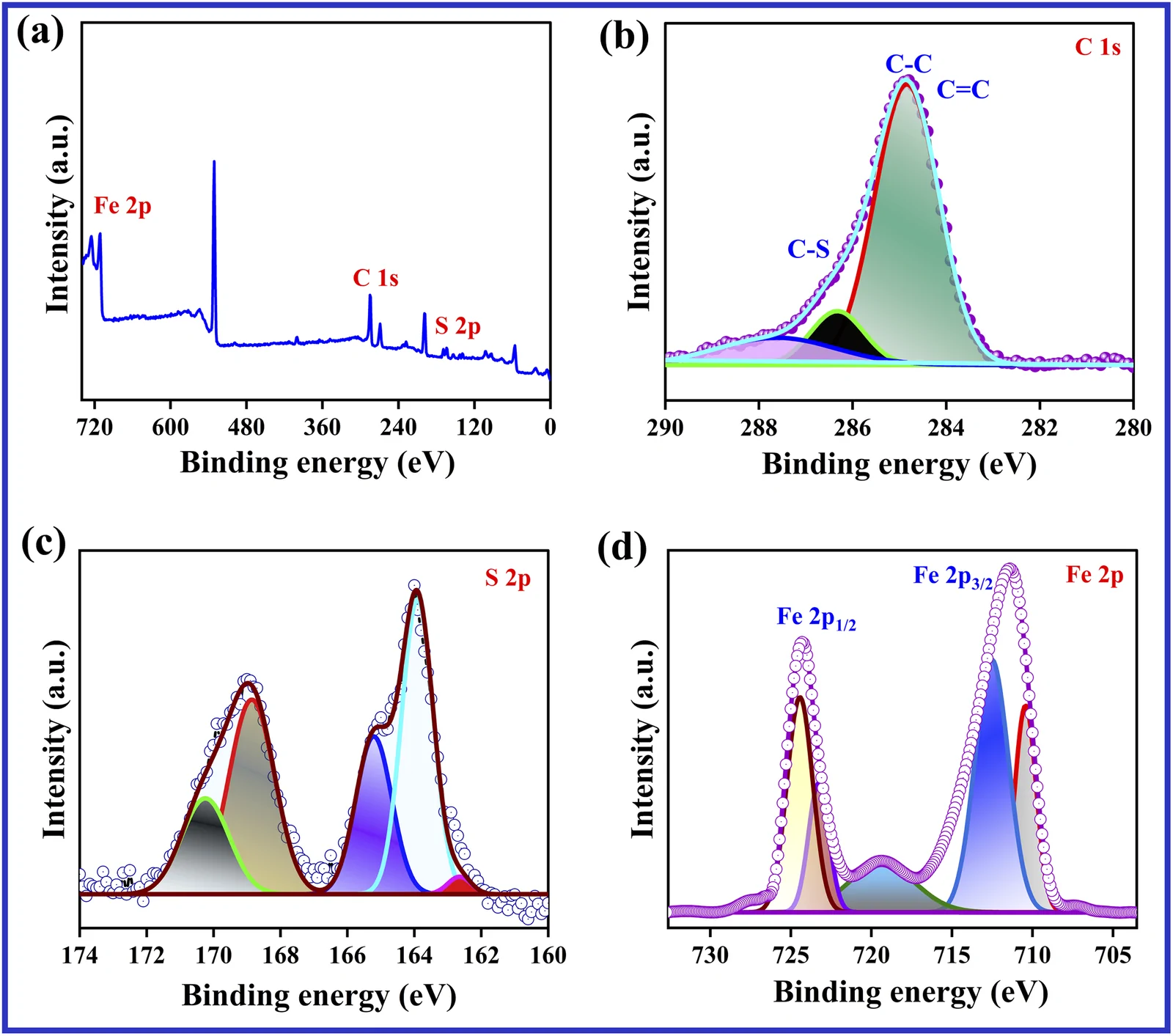
(a) XPS survey spectrum of FeS_2_@PTh. High-resolution XPS spectra of (b) C 1s, (c) S 2p and (d) Fe 2p.

### 3.3 Surface morphology and elemental composition analyses

Fig. 4 presents the morphology and interfacial structure of polythiophene, FeS_2_, and the FeS_2_@PTh heterostructure, as analysed by FE-SEM. In Fig. 4(a–c), polythiophene is observed as irregularly shaped particles with a tightly packed and relatively smooth surface exhibiting slight roughness. Fig. 4(d–f) displays the microstructure of FeS_2_, characterised by irregular, plate-like nanoplates that are stacked and partially packed. Furthermore, Fig. 4(g–i) reveals that the composite contains densely distributed FeS_2_ nanoparticles embedded within the polythiophene matrix, forming a well-integrated FeS_2_@PTh heterostructure. Aggregation of FeS_2_@PTh is attributed to strong π–π interactions between polymer chains, which act as a conductive matrix, facilitating FeS_2_ dispersion and preventing agglomeration. Strong interfacial interactions between FeS_2_ and PTh are evident, enhancing charge transfer and improving photocatalytic performance. Elemental mapping and energy-dispersive X-ray spectroscopy (EDS) (Fig. 4(j–n)) indicate atomic percentages of 31.40%, 30.34%, and 38.26%, confirming the presence of iron, sulphur, and carbon in the composite.

**Fig. 4:**
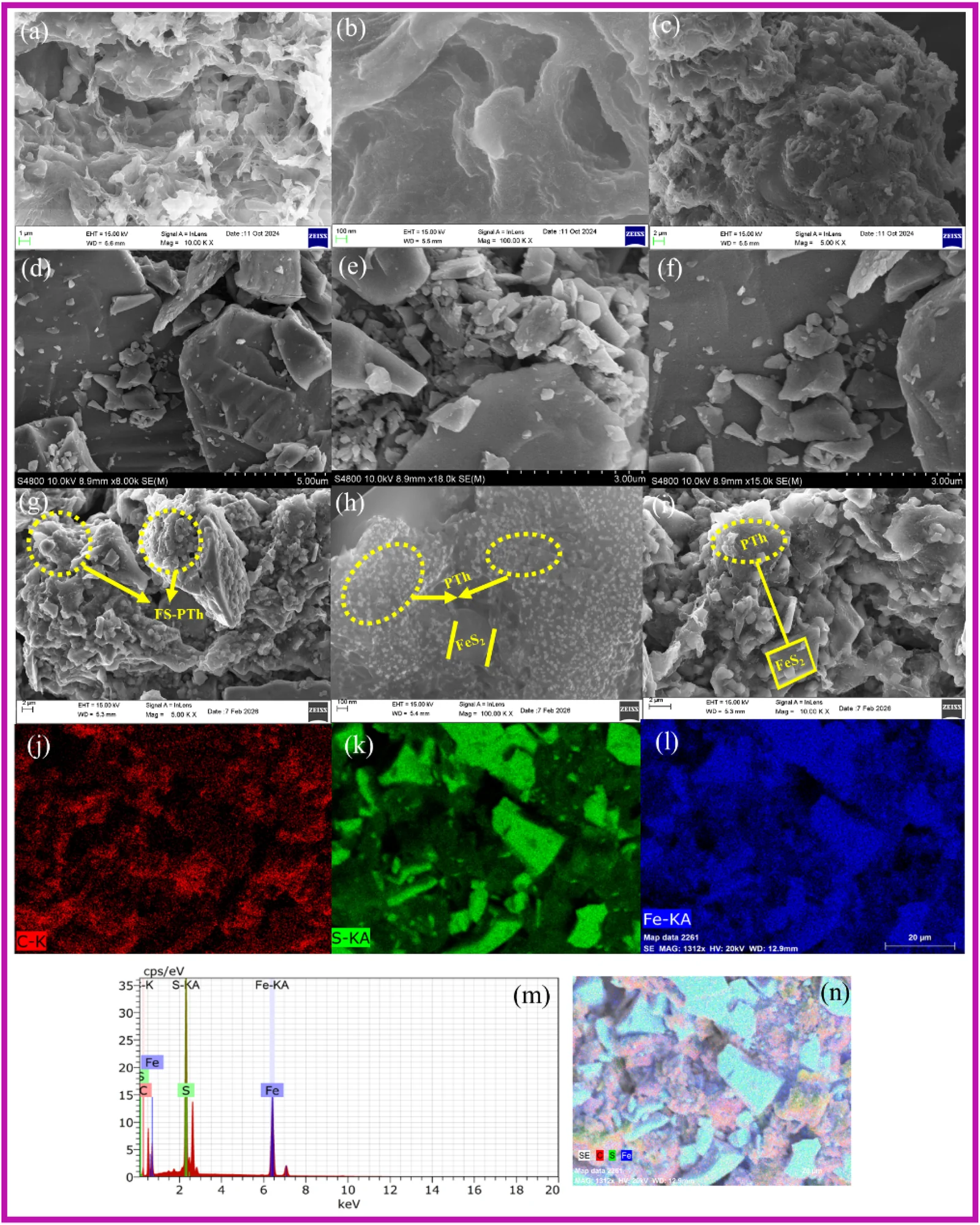
FE-SEM images of (a–c) polythiophene, (d–f) FeS_2_ and (g–i) FeS_2_@PTh nanohybrid. (j–l) Elemental mapping images, (m) EDS and (n) overall mapping view.

HR-TEM images of FeS_2_@PTh are shown in Fig. 5(a–h), which clearly show agglomerated particles with tight contact between FeS_2_ and PTh in the heterostructure. The HR-TEM lattice fringes with a *d*-spacing of 0.378 nm correspond to the (111) plane of FeS_2_, confirming its crystalline nature. The selected-area electron diffraction (SAED) pattern of the heterostructure is shown in Fig. 5(i), indicating a polycrystalline nature. The rings (Fig. 4(h)) are indexed to the (111), (200), (210), (211), and (220) planes of cubic FeS_2_, confirming a well-defined crystal nature. These results collectively demonstrate the successful formation of a heterostructure. As shown in Fig. 6(a–c), the particle size and distribution behaviour were analysed by a dynamic light scattering particle sizer. Fig. 6(a) shows that pure polythiophene exhibited a larger hydrodynamic size of 480.35 nm, which is attributed to the polymer chain formation of soft agglomerates due to intrinsic structural flexibility. Fig. 6(b) shows that FeS_2_ NPs have an average hydrodynamic diameter of 93.32 nm, with a highly heterogeneous size dispersion and a small average size. However, the composite exhibited an intermediate dynamic size of 315 nm, with increased particle size compared to pristine FeS_2_, confirming the successful coating of PTh on the NPs surface, while reducing the size relative to PTh. This indicates improved dispersion behaviour upon polymer incorporation, which could be observed as a trend further supporting the formation of the composite.^39^

**Fig. 5:**
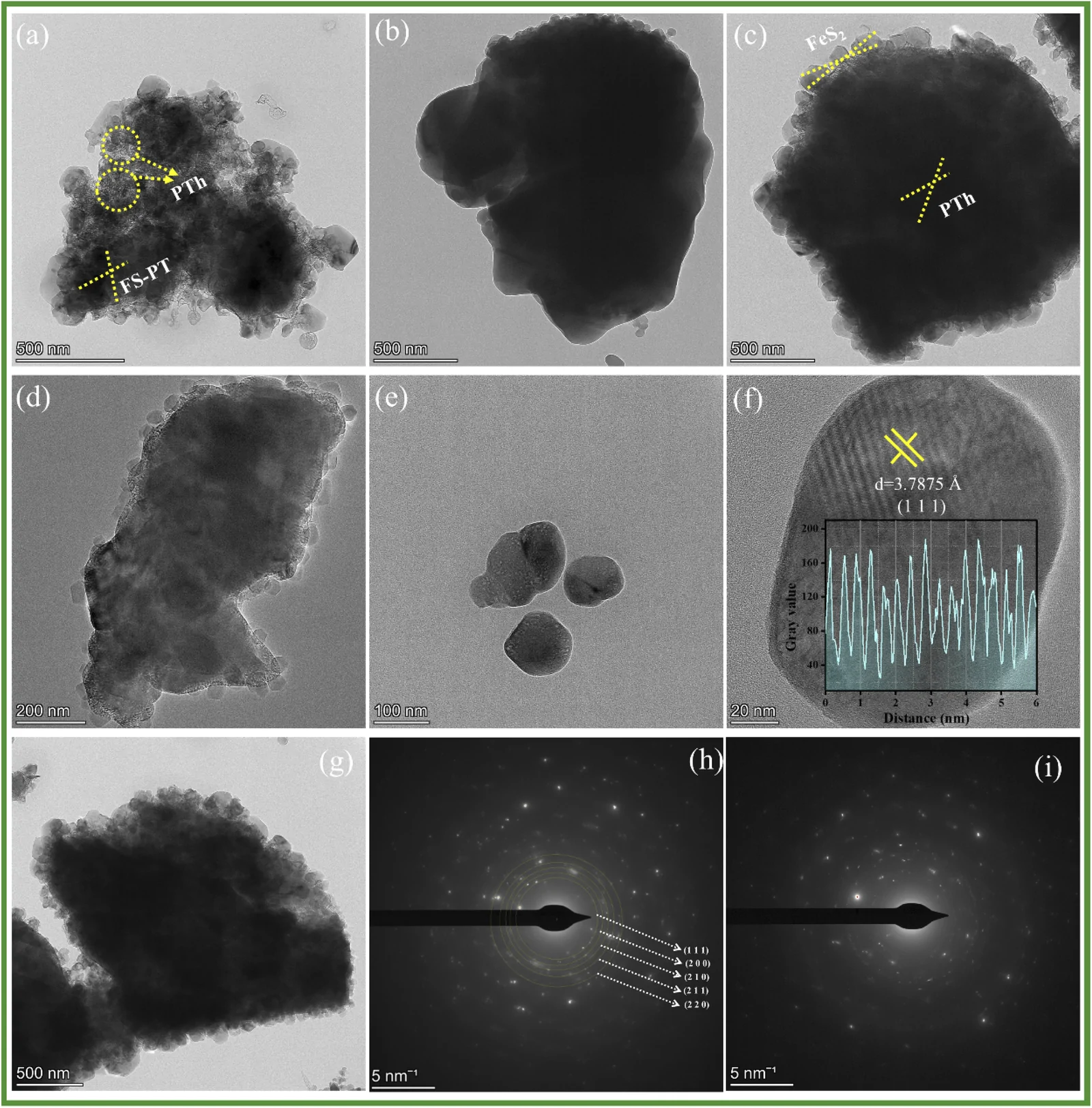
(a–g) HRTEM images and (h and i) SAED patterns of FeS_2_@PTh.

**Fig. 6:**
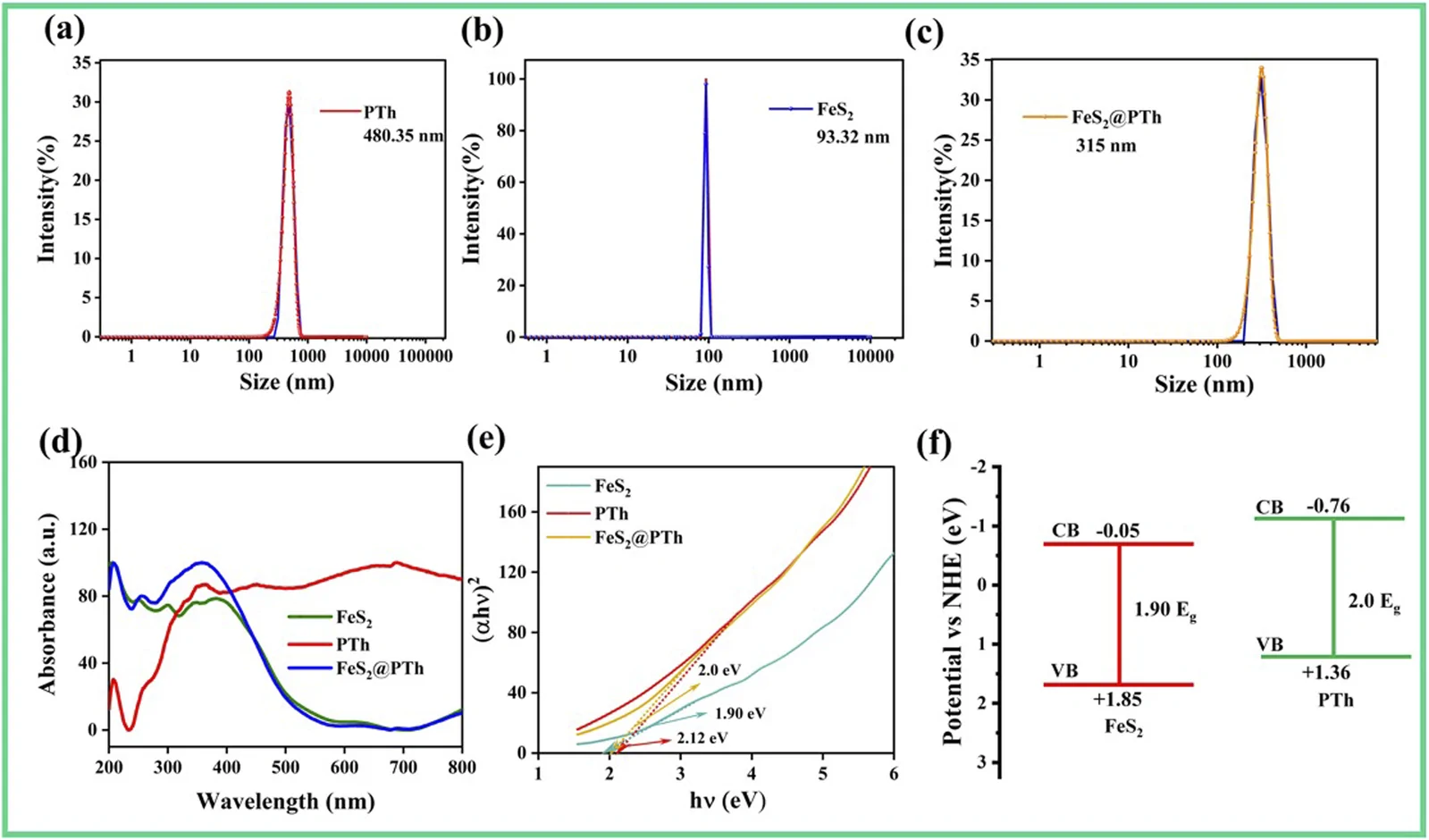
DLS particle sizes of (a) PTh, (b) FeS_2_ and (c) FeS_2_@PTh. (d) DRS-UV spectra, (e) Tauc plots and (f) band diagrams.

### 3.4 Optical analysis

The optical properties of FeS_2_@PTh were studied using UV-visible diffuse reflectance spectroscopy (UV-DRS). As shown in Fig. 6(d), the spectrum of pure polythiophene exhibited a broad shoulder peak at 360 nm, which could be attributed to π–π orbital transitions within the polymer.^23^ FeS_2_ NPs exhibit 200–800 nm with good absorption.^40^ However, two absorption peaks at 360 nm and 208 nm in FeS_2_@PTh could arise from PTh and FeS_2_, respectively, and are notable in the spectrum of the nanocomposite. In FeS_2_@PTh, a higher absorption intensity is shown; this change is attributed to the interaction and efficient charge transfer between pyrite and the conjugated polymer, leading to improved visible-light absorption and charge-transfer kinetics. In Fig. 6(e), the Tauc relation is used to determine their bandgap as follows:^41^

1 *αhν* = *A*(*hν* − *E*_g_)^*n*^,

where *h*, *ν*, *E*_g_, *A* and *α* represent the Planck constant, light frequency, optical bandgap energy, constant and absorption coefficient, respectively. The Planck constant is the proportionality constant related to the material *vs.* energy bandgap. The bandgaps of PTh, FeS_2_ and FeS_2_@PTh are 2.12, 1.90 and 2.0 eV, respectively (Fig. 6(f)). In addition, the valence band and conduction band functions were estimated using the following equations, respectively:^42,43^

2 *E*_VB_ = *X* − *E*_e_ + 0.5*E*_g_,

3 *E*_CB_ = *E*_VB_ − *E*_g_,

where *X* is the electronegativity of the semiconductor, *E*_g_ is the forbidden band gap (PTh, FeS_2_ and FeS_2_@PTh), and *E*_e_ is the energy of a free electron (4.5 eV on the hydrogen scale).

### 3.5 Photocatalytic degradation of TC and CIP

Degradation of TC and CIP was performed through the photocatalytic process under 160 W visible-light irradiation. Fig. 7(a and b) shows the time-dependent absorbance plot of TC and CIP in the presence of FeS_2_@PTh. Fig. 7(c and d) shows the *C*/*C*_0_ plot *versus* time-dependent concentration, and the respective linear fit values for FeS_2_, PTh, and FeS_2_@PTh are shown in Fig. 7(e and f). The data show a good linear fit with high *R*^2^ values, consistent with the Langmuir–Hinshelwood kinetics described by the following pseudo-first-order function:

4 −ln(*C*_0_/*C*_*t*_),

where *k* is the rate constant, *C*_*t*_ represents TC and CIP removal at time *t*, and *C*_0_ is the initial concentration at time zero. As shown in Fig. 7, the FeS_2_@PTh hybrid exhibited higher degradation performance, removing 83% of TC (*k* = 1.422 × 10^−2^ min^−1^) and 85% of CIP (*k* = 1.687 × 10^−2^ min^−1^). The respective rate plots show higher *k* values compared to pristine materials. The FeS_2_@PTh heterostructure with different catalyst dosages, namely, FPT-10, FPT-30, and FPT-50 mg L^−1^, exhibited TC degradation efficiencies of 28.78% (*k* = 2.92 × 10^−3^ min^−1^), 83% (*k* = 1.422 × 10^−2^ min^−1^), and 74% (*k* = 1.028 × 10^−2^ min^−1^), respectively. Among these, FPT-30 showed the highest photocatalytic performance due to the optimal availability of active sites and effective charge separation. However, further increasing the catalyst dosage to FPT-50 led to a slight decrease in degradation efficiency, which may be attributed to particle agglomeration and light scattering effects that reduce active surface area and photon penetration, respectively. Lower catalyst loading results in fewer active sites and a smaller degradation rate. However, beyond the optimum dosage, further increases in catalyst amount can lead to scattering effects that reduce light penetration, thereby lowering degradation efficiency.^6,44^ Therefore, the optimum catalyst dosage is essential for the removal of TC and CIP.

**Fig. 7:**
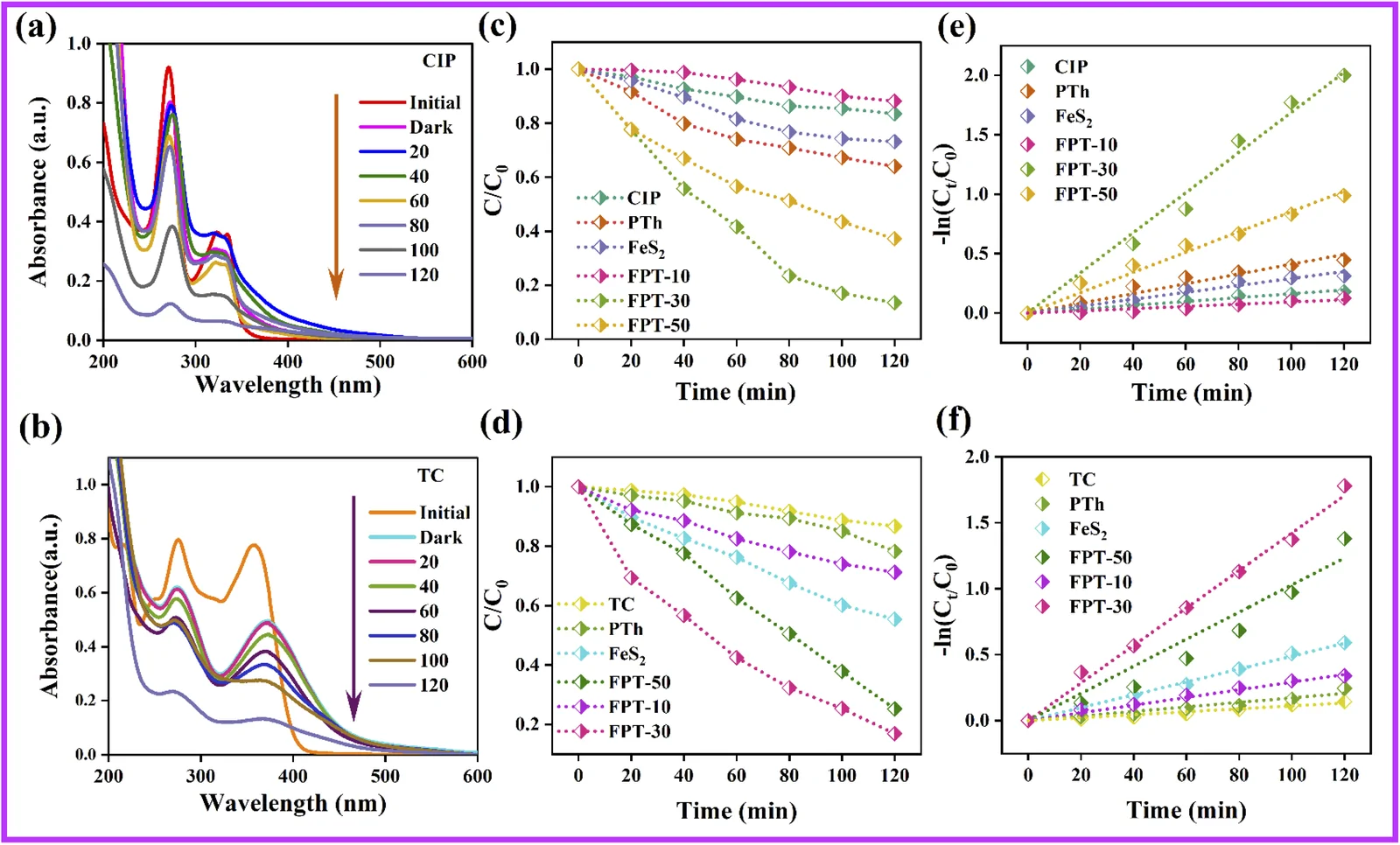
UV-visible absorbance spectra of (a) CIP and (b) TC removal with the FeS_2_@PTh nanohybrid. (c and d) *C*/*C*_0_*versus* irradiation time (min) and (e and f) pseudo-first-order kinetics plots.

As shown in Fig. 8(a–d), the TC and CIP removal rates (*k* values) were higher in the composite system, which may be due to synergistic effects, including electron–hole accumulation mediated by an increased number of active sites that break down TC and CIP molecules. Fig. 9(a and b) further verifies the repeatability of the catalyst material. Under the same ideal conditions, *C*/*C*_0_*versus* irradiation time shows the optimum dosage, with negligible degradation and removal of TC and CIP, and their corresponding *k* rates are similar to the triplicate condition. Fig. 9(c) displays the overall experimental efficiency.

**Fig. 8:**
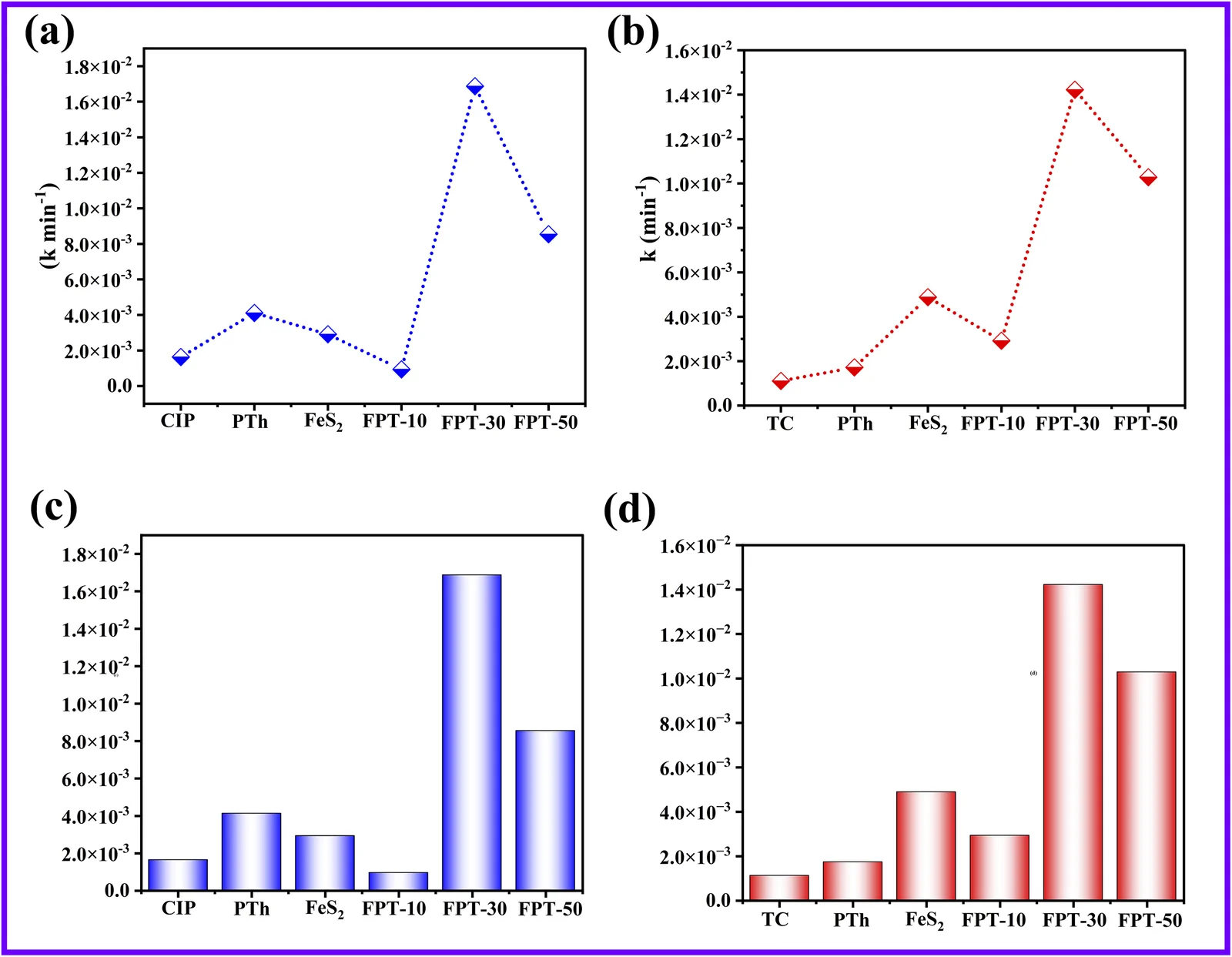
(a–d) Rate constant plots against CIP and TC.

**Fig. 9:**
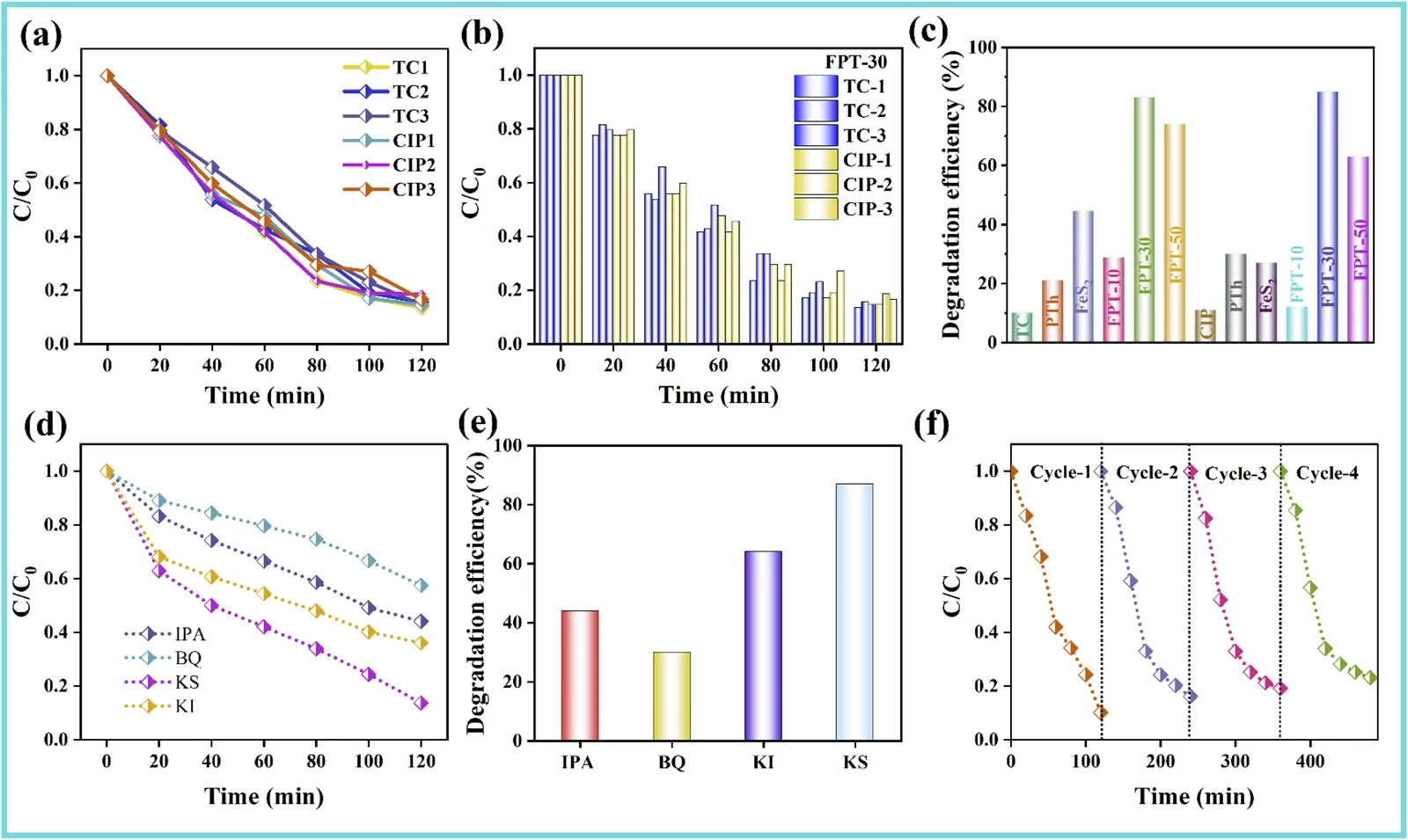
(a and b) Repeatability tests for the removal of TC and CIP by FeS_2_@PTh. (c) Overall efficiency (%). (d and e) Scavenger studies and (f) stability study.

Fig. 9(d and e) depicts the results of the scavenger analysis, which was conducted to determine the dominant reactive species in the photocatalytic degradation of TC, the analysis was performed using isopropyl alcohol (IPA), benzoquinone (BQ), potassium iodide (KI), and potassium persulfate (KS) as quenchers for ˙OH, ˙O_2_^−^ radicals, h^+^ and e^−^, respectively. Upon addition, BQ and IPA significantly suppressed degradation efficiency, reducing TC removal from 83% to 30% and 43%, respectively, indicating that these radicals are the principal reactive species. Inclusion of the h^+^ scavenger increased the degradation rate by 68%. It could act as a secondary reactive species, while in the presence of KS, a moderate reduction of 87% for TC was observed. This factor plays a crucial role in the degradation and removal of TC; the generated hydroxyl group promotes O_2_ absorption and activation to form ˙O_2_^−^, and holes formed by photogeneration are directly oxidised.^45^ However, as shown in Fig. 9(f), the stability of the FeS_2_@PTh catalyst is maintained by the removal of TC, as monitored in four consecutive cycles, with 79% after the 4th cycle. It maintains its form as a synergistic catalyst, showing good stability performance. Moreover, Table 1 compares the photodegradation efficiency of the FeS_2_ and PTh based photocatalysts for the TC and CIP pollutants with those reported in previous literature. The comparative results suggest that the prepared FeS_2_@PTh heterostructured composite materials have enhanced photocatalytic activity compared with pristine and other materials.

**Table 1:** Some reported FeS_2_- and PTh-based photocatalysts for the degradation of TC and CIP

Photocatalyst	Pollutant	Light source	Reaction time (min)	Efficiency (%)	Ref.
g-C_3_N_4_@FeS_2_	TC	300 W Xe lamp	40	97.24	46
FeS_2_/CN	TC	300 W Xe lamp	60	96	47
E-MoS_2_/FeS_2_	TC	300 W xenon lamp	40	90.6	48
FeS_2_/g-C_3_N_4_	TC	300 W xenon lamp	180	83.5	49
FeS_2_@Co_3_O_4_–C/PMS	TC	Visible	20	97.2	50
Fe_2_(MoO_4_)_3_/Cd_0.5_Ni_0.5_S	TC	Visible	70	98	51
PTh/S–CN	TC	500 W xenon lamp	60	80.9	52
WS_2_@PTh	TC	UV light	240	90	53
	CIP			75	
MoS_2_@polythiophene	TC	160 W halogen lamp	240	90	27
	CIP		240	83	
FeS_2_@PTh	TC	160 W halogen lamp	120	83	Present work
	CIP			85	

### 3.6 Plausible degradation mechanism

To further understand the mechanism behind the working FeS_2_@PTh, the band level positions were estimated using the electronegativity-based approach. With *X* (FeS_2_ = 5.40 eV)^54^ and the hydrogen scale-free electron energy, the calculated band positions (*E*_CB_, −0.05 eV for FeS_2_ and −0.76 eV for PTh *vs.* NHE) and the CB of the composite (*E*_VB_ was estimated at +1.85 eV and +1.35 eV *vs.* NHE) were obtained, and shown in Fig. 10. In Fig. 6, the band potential was estimated from UV-DRS and the values for FeS_2_ (1.90 eV) and PTh (2.12 eV). The Mott–Schottky measurements provide further information on the semiconductor behaviour, where negative and positive slopes were assigned to the p-type and n-type semiconductors of FeS_2_ and PTh, respectively, as provided in SI (Fig. S1). This allows us to understand the formation of a direct Z-scheme heterojunction that facilitates efficient charge separation. In the heterostructure photocatalyst, upon visible light irradiation of PTh and FeS_2_, an electron–hole pair is formed. In the conduction band of FeS_2_, photoexcited electron transferred from the PTh matrix participate in parallel redox reactions, converting dissolved oxygen into superoxide radicals, while reducing Fe^3+^ species to Fe^2+^.^45^ The photogenerated electrons in the conduction band of FeS_2_ recombine with holes in the valence band of PTh. Meanwhile, the remaining electrons in the conduction band of PTh directly reduce dissolved oxygen to form (˙O_2_^−^), subsequently generating ˙OH radicals, whereas holes in the VB of FeS_2_ participate in direct oxidation.^45^ In contrast, the favourable band alignment of the CB of PTh (−0.76 eV) is more negative than the potential (−0.33 eV *vs.* NHE), enabling the formation of superoxide radicals, as confirmed by the trapping results.^54,55^ However, in the valence band of FeS_2_ (+1.85 eV), ˙OH formation occurs indirectly *via* H_2_O_2_ intermediates.^56^ Meanwhile, OH is an active reactive species; both TC and CIP degrade through attack by ˙O_2_^−^ and ˙OH, ultimately leading to mineralisation into CO_2_ and H_2_O.^44^

**Fig. 10:**
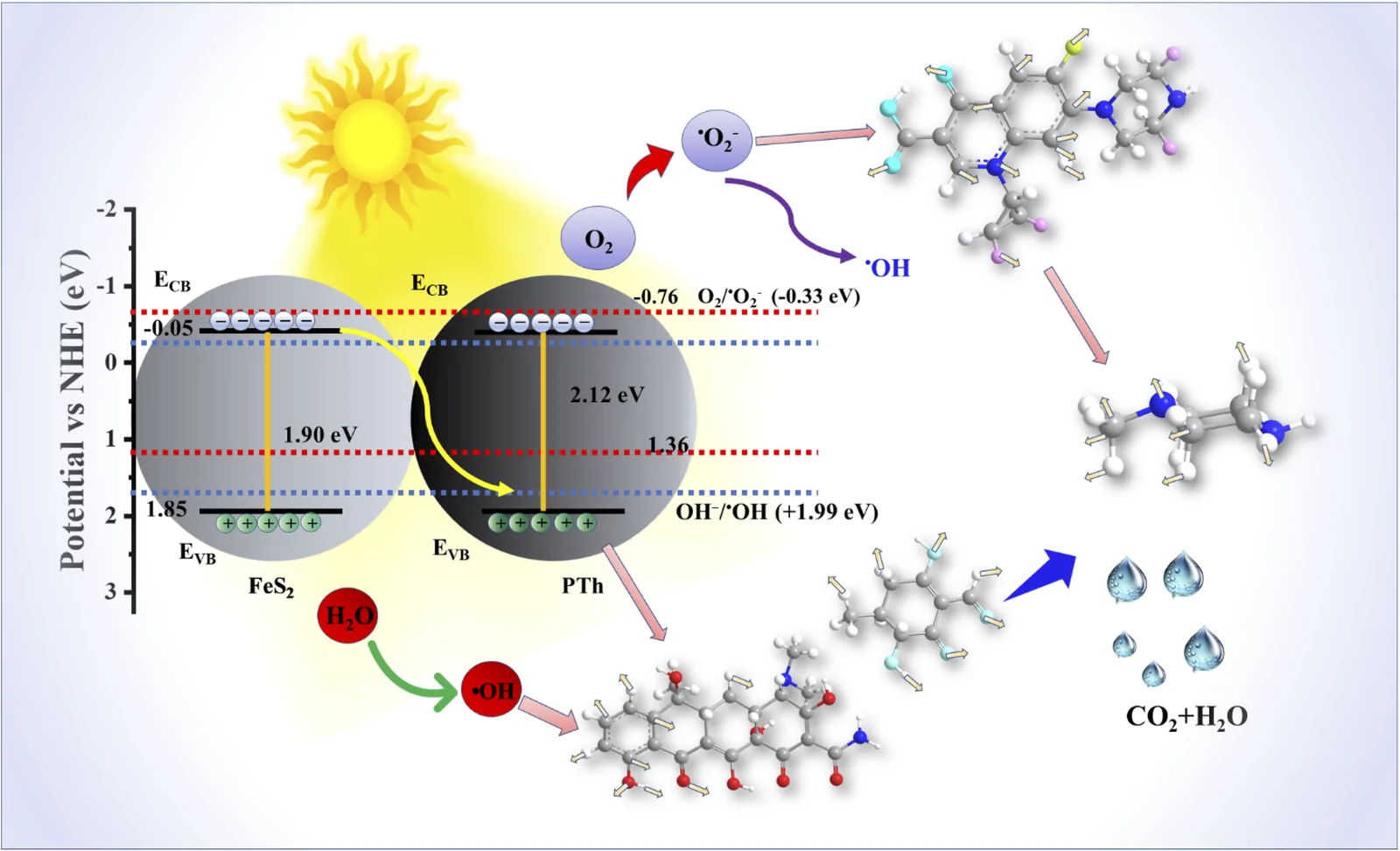
Plausible degradation process of TC and CIP.

## 4. Conclusions

A facile and effective strategy was used to synthesise the heterostructured FeS_2_@PTh photocatalyst, and its photocatalytic performance was evaluated. The optimised photocatalyst achieves the removal of TC (83%, *k* = 1.422 × 10^−2^ min^−1^) and CIP (85%, *k* = 1.687 × 10^−2^ min^−1^) under visible-light. Optical analysis revealed bandgap energies of PTh, FeS_2_, and the FeS_2_@PTh composite of 2.12, 1.90, and 2.0 eV, respectively. The intermediate optical bandgap of FeS_2_@PTh compared with the pristine components suggests effective interfacial electronic interaction between FeS_2_ and PTh. Radical trapping experiments were conducted, which demonstrated that the primary oxidants were hydroxyl radicals (˙OH), superoxide radicals (˙O_2_^−^) and secondary species of h^+^ (tested with potassium iodide) and e^−^ (tested with potassium persulfate). Repeatability and reusability studies over four cycles for TC revealed negligible degradation in efficiency (79%). A direct Z-scheme pathway was proposed involving conventional electron transfer and charge separation kinetics. The optimised photocatalyst plays a synergistic role in the degradation of TC and CIP, and it offers an eco-friendly and efficient approach to wastewater treatment.

## Conflicts of interest

There are no conflicts to declare.

## Supplementary Material

NA-OLF-D6NA00587J-s001

## Data Availability

The data supporting this article have been included as part of the supplementary information (SI). Supplementary information: photoelectro-chemical measurements and post-characterization results. See DOI: https://doi.org/10.1039/d6na00587j.
