# Supplementary material for: Visible-light driven degradation of tetracycline and ciprofloxacin using a Z-scheme FeS2@polythiophene heterostructure photocatalyst
Source: Nanoscale Adv. 2026 Sep 14;8(22):6705–17. doi: 10.1039/d6na00587j (PMC13648040; doi:10.1039/d6na00587j)
Supplement: Supplementary file 1 [file NA-008-D6NA00587J-s001.pdf]

Supplementary Information (SI) for Nanoscale Advances.
This journal is © The Royal Society of Chemistry 2026

Supporting information

Visible light driven degradation of Tetracycline and Ciprofloxacin using a Z-scheme FeS_2@Polythiophene heterostructure Photocatalyst

Maruthasalam Pannerselvam[1,2], Vadivel Siva[1,2*], Abdul samad Shameem[2,3], Anbazhagan Murugan[4], Krishnasamy Padmavathi[1,2,] Murugan Dineshkumar[1,2], Shaik Gouse Peera[5]

1. Photoelectrochemical measurements

Electrochemical impedance spectroscopy (EIS) and photocurrent measurements and Mott-Schottky analysis were carried out by an electrochemical analyser (CHI 660F in a three-electrode system in 0.1 M Na_2SO_4, where Ag/AgCl was used as a reference electrode (1 ×1 cm^2) with a Pt wire as a counter electrode. Material-coated FTO was the working electrode. The working electrode was prepared via the drop-casting method. 10 mg of material was dispersed in a mixture of 60 µl Nafion, 100 µl DI water, and 140 µl of 99% ethanol, followed by ultrasonication for 30 min.

1.1 Mott Schottky, Photocurrent and EIS

The semiconductor type and the flat-band potential of the photocatalysts were investigated using Mott–Schottky analysis based on the capacitance–voltage (C_2–V) relationship obtained from electrochemical measurements. As shown in (Fig. S1a & b) the Mott–Schottky plots of FeS_2 and PTh exhibit positive and negative slopes, confirming their p type n type semiconductor characteristics. The flat-band potentials E_{fb} of FeS_2 and PTh were determined to be −0.39 and −0.43 V (vs Ag/AgCl, pH = 7), respectively [1,2]. The conduction band potentials were converted to the normal hydrogen electrode (NHE) scale using the following equation [3],

$$E_{NHE} = E_{Ag/AgCl} + 0.197,$$

Accordingly, the conduction band potentials of FeS_2 and PTh were estimated to be -0.193 and 0.627 V (vs NHE), respectively. Photogenerated charge carrier separation and transport properties were evaluated by photocurrent measurements, as shown in (Fig. S1c). During the light on/off cycle, photocurrent response significantly increases and then decreases, indicating

a reduced recombination rate and enhanced movement of charge carriers. However, FeS_2@PTh shows an excellent photocurrent response compared to pure and pristine materials. This can undoubtedly be attributed to heterojunction formation between FeS_2 and PTh. As shown in (Fig. S1d). Furthermore, electrochemical impedance spectroscopy (EIS) analysis was used to understand the charge transfer characteristics of the photocatalyst. The Nyquist plots are provided FeS_2@PTh composite exhibits a substantially smaller arc compared to pristine materials. The charge transfer resistance is inversely connected to the arc radius of the Nyquist plot. FeS_2@Pth exhibits a smaller semicircle arc, indicating improved charge transfer and migration at the electrode/electrolyte interface.

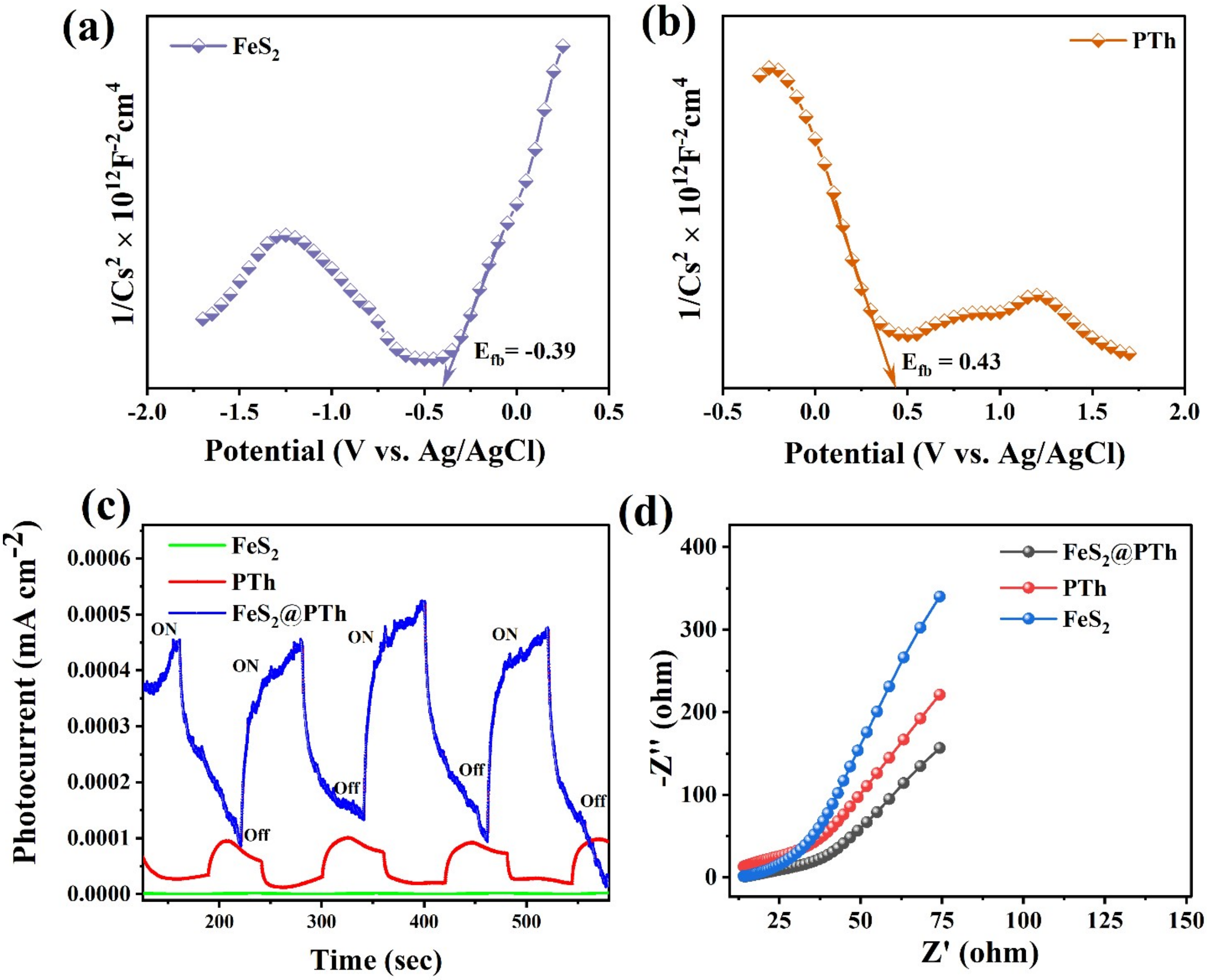

Figure S1. (a-b) Mott Schottky experiments c) photocurrent and d) EIS

2. FTIR analysis

The FT-IR spectra were examined in the range of 4000–550 cm^{-1}. Fig. S2 displays prominent absorption bands at 1618, 1314, 1192, 1110, 669 and 623 cm^{-1}. The band at 1618 cm^{-1} was assigned to the C=C and C–C stretching of the thiophene ring, while the bands at 1314 and 1192 cm^{-1} were attributed to C–C vibrations coupled with C–H in-plane deformation of the thiophene ring [4]. The weak bands observed at 669 and 623 cm^{-1} are attributed to C–S and C–S–C ring deformation vibrations, characteristic of the thiophene unit in polythiophene [4-6]. The FeS_2 (NPs) exhibited a broad band centred at 3419 cm^{-1} and another at 1620 cm^{-1}, which could be assigned to the O–H stretching and bending modes of water, respectively. [7]. In the FeS_2@PTh composite, noticeable shifts in several characteristic peaks are observed. These slight peak shifts indicate interfacial interactions, chemical compatibility, and possible charge transfer between the PTh and FeS_2.

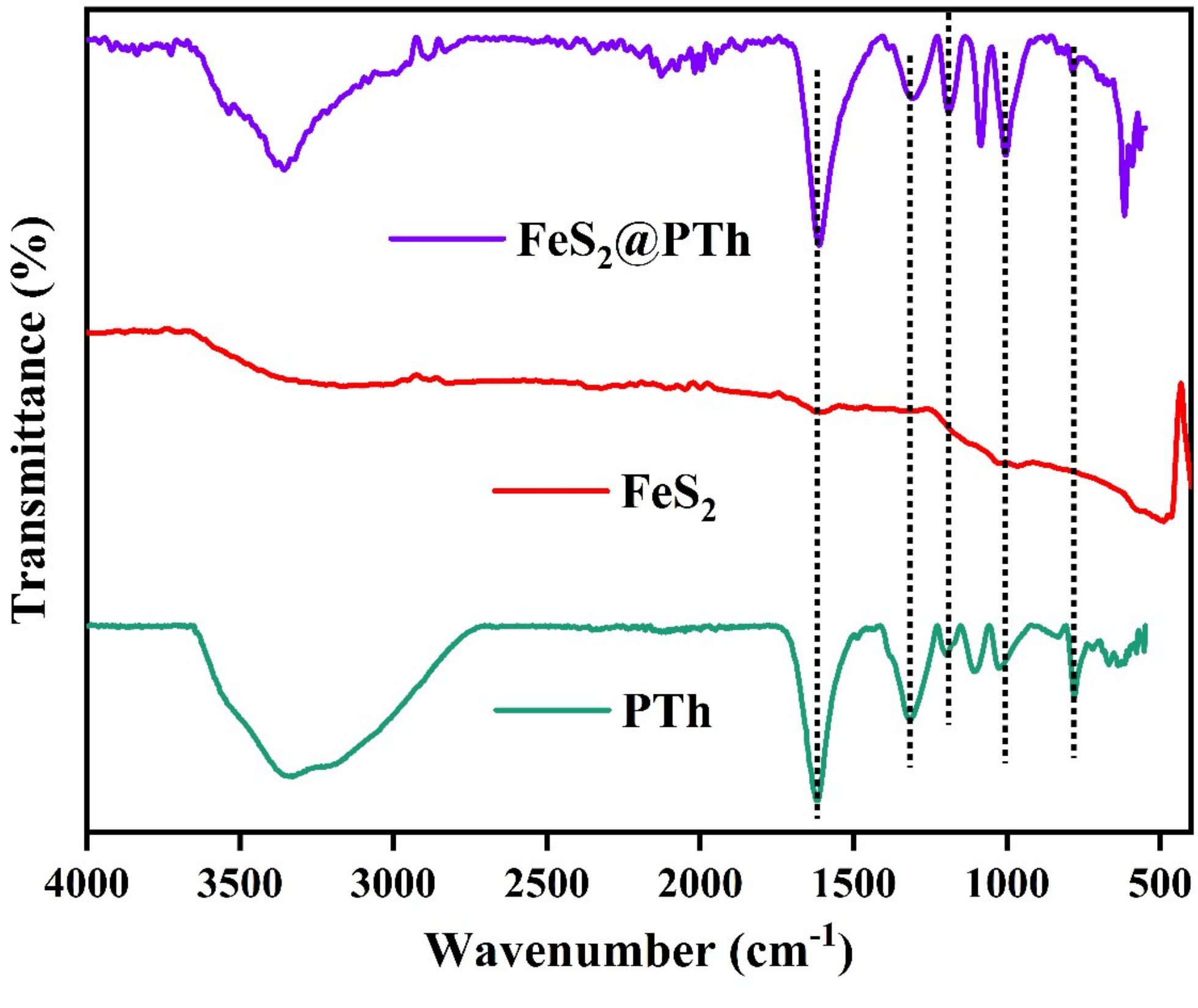

Figure S2. FT-IR spectra of PTh, FeS_2 and FeS_2@PTh

3. **Post-catalyst analysis**

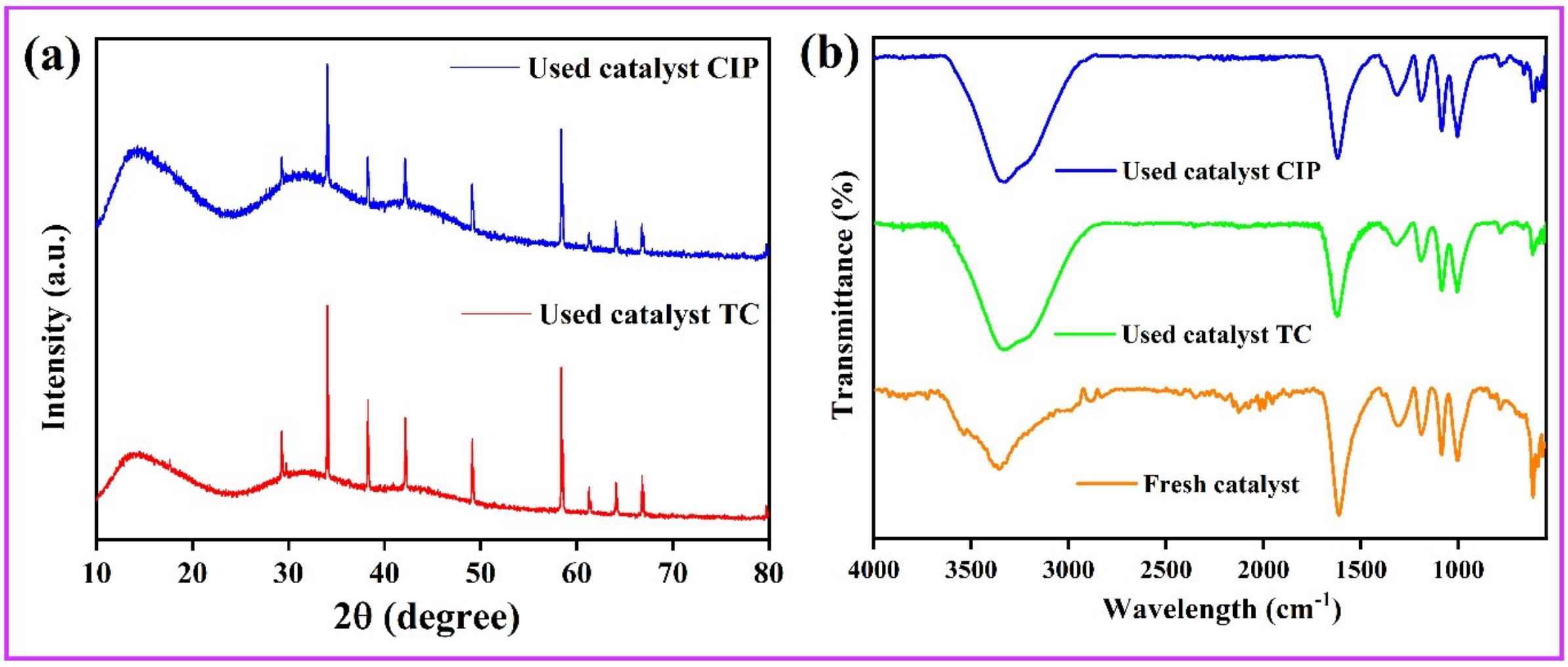

Figure S3. a) XRD and b) FTIR analysis post-cycle experiment

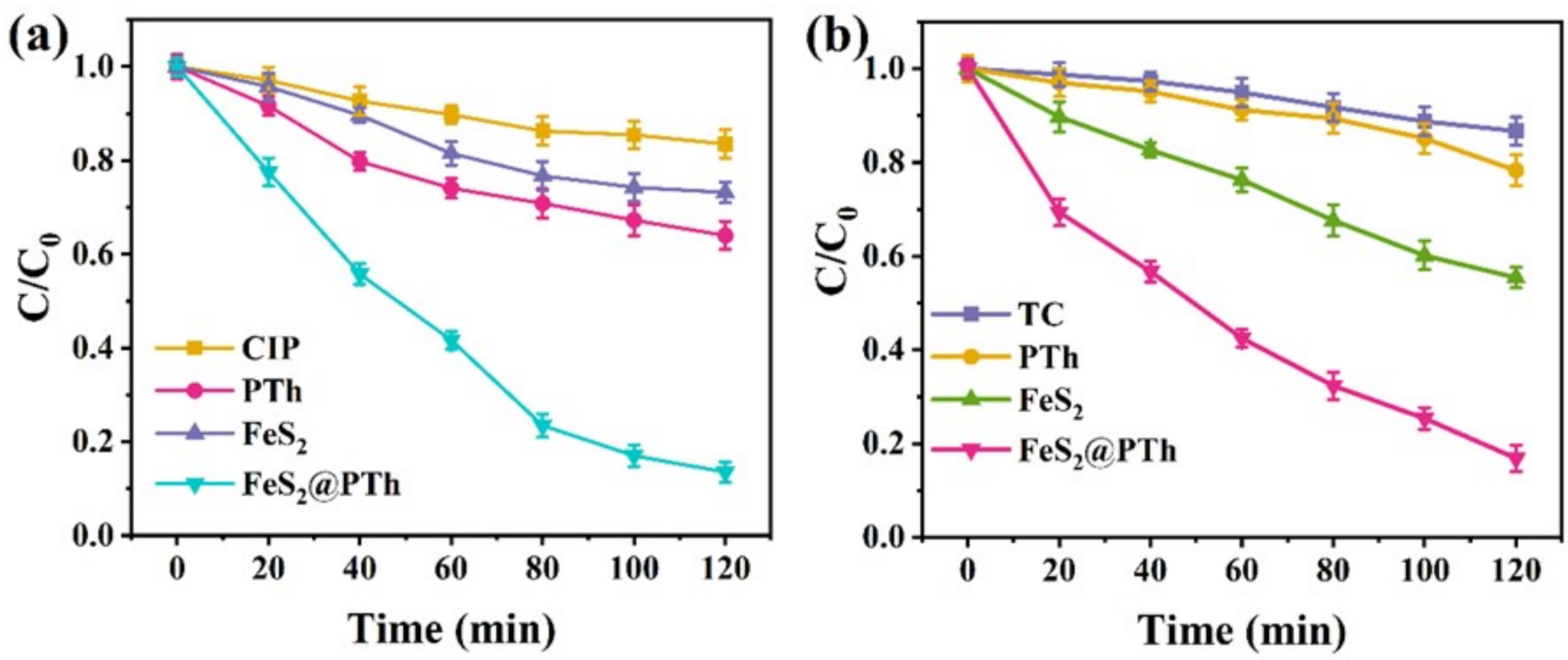

Figure.S4. Triplicate experimental results with error bars showing the C/C0 degradation profiles of the photocatalytic system.

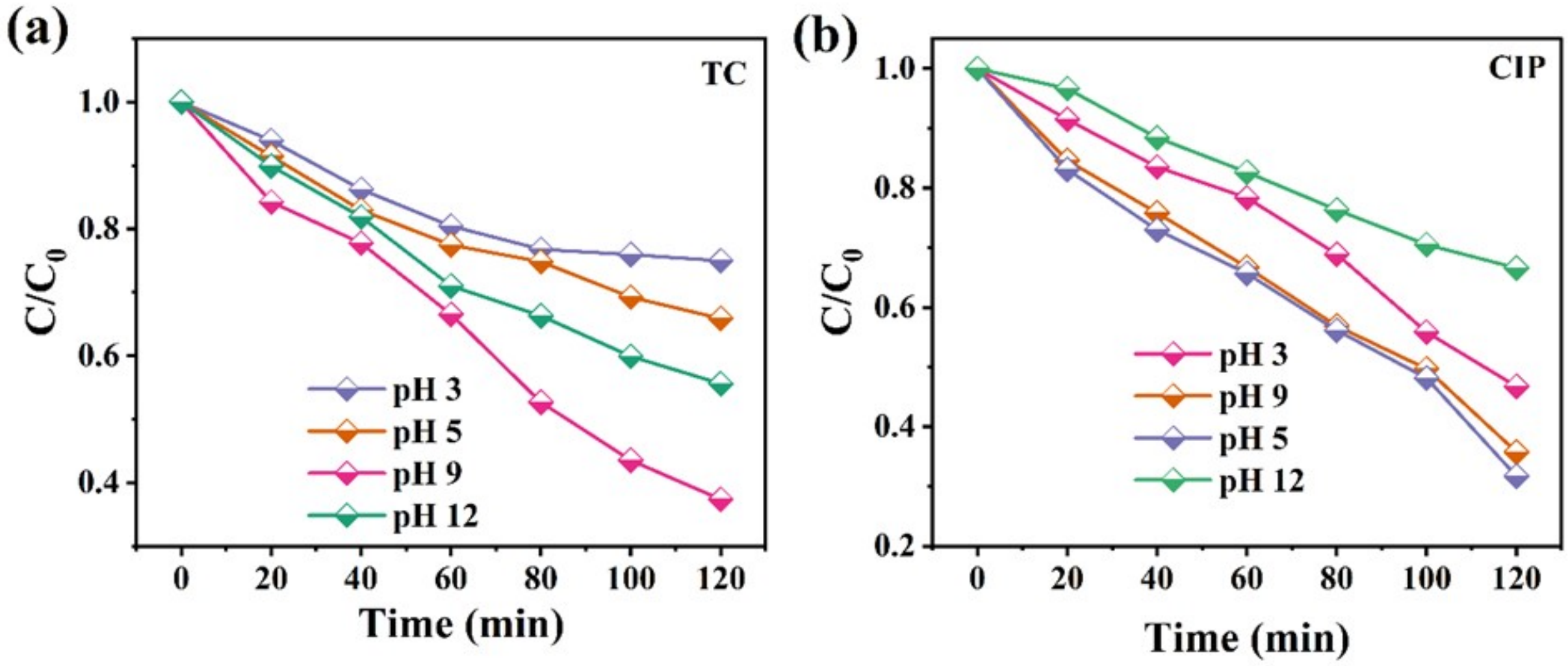

Figure S5. Different pH conditions: a) TC and b) CIP

References

[1] C. Ouyang, X. Quan, C. Zhang, Y. Pan, X. Li, Z. Hong and M. Zhi, Direct Z-scheme $ZnIn_2S_4$@MoO_3 heterojunction for efficient photodegradation of tetracycline hydrochloride under visible light irradiation, *Chem. Eng. J,* 2021, 424, 130510, DOI:10.1016/j.cej.2021.130510.

[2] M. Zhang, J. Zhang, Highly selective NH_3 sensor based on MoS_2/WS_2 heterojunction, *Nanomaterials,* 2023, 13, 1835, DOI: 10.3390/nano13121835.

[3] T. Sun, L. Yang, Y. Huang, Y. Zhu, H. Yang, L. Bai, D. Wei, K. Yin, W. Wang, H, Chen, Z-scheme $BiOBr/WS_2$ heterojunction with integrated photocatalytic degradation and photothermal water evaporation for effective dye wastewater purification, *J. Alloys Compd,* 2025, 1010, 177482, DOI: 10.1016/j.jallcom.2024.177482.

[4] A. Husain, S. Ahmad, F. Mohammad, thermally stable and highly sensitive ethene gas sensor based on polythiophene/zirconium oxide nanocomposites, *Mater. Today Commun,* 2019, 20, 100574, DOI: 10.1016/j.mtcomm.2019.100574.

[5] A. Husain, S. Ahmad, S.A. Alqarni, S.J. Almehmadi, M.A. Yatoo, F. Habib, M.U. Shariq, Conductive polythiophene/graphitic-carbon nitride nanocomposite for the detection of ethanol mixing in petrol, *RSC Adv,* 2023, 13, 12080–12091, DOI:10.1039/d3ra01039a.

[6] M. Aydın, S.R. Tokgoz, M.A. Çarpan, H. Delen, A. Peksoz, Electrochemically synthesized polythiophene molecules: Effect of layer thickness on electrochromic performance and photocurrent response, *Surf. Interfaces,* 2025, 106974, DOI: 10.1016/j.surfin.2025.106974.
